# Lobar-level radiomic clustering reveals background lung changes associated with lung cancer risk: a new perspective for early screening

**DOI:** 10.1186/s13244-026-02328-y

**Published:** 2026-06-20

**Authors:** Yihuan Wang, Chen Zhu, Zhenzhen Lu, Xianglan Zhou, Chengting Lin, Yuwei Li, Liting Shi, Lili Wu, Hongxia Ma, Meng Zhu, Jia Chen, Junwei Lv, Lingying Zhu, Lingbin Du, Chen Ji, Honglun Ren, Enyu Wang, Lei Shi

**Affiliations:** 1 Department of Radiology, Zhejiang Cancer Hospital, Hangzhou Institute of Medicine (HIM), Chinese Academy of Sciences, Hangzhou, Zhejiang China; 2 Department of Cancer Prevention, Zhejiang Cancer Hospital, Hangzhou Institute of Medicine (HIM), Chinese Academy of Sciences, Hangzhou, Zhejiang China; 3 Department of Radiology Imaging, Taizhou Cancer Hospital, Taizhou, China; 4 Department of Epidemiology, Center for Global Health, School of Public Health, Nanjing Medical University, Nanjing, China; 5 Faculty of Health Sciences, University of Macau, Macao, China; 6 Shanghai Juillet AI Lab, Shanghai, China; 7 BEIJING DEEPWISE&LEAGUE OF PHD TECHNOLOGY CO.LTD, Beijing, China

**Keywords:** Radiomics, Lung cancer, Unsupervised clustering, Field cancerization, Risk stratification

## Abstract

**Background:**

The concept of field cancerization highlights spatially diffuse, pre-malignant changes in carcinogen-exposed lung tissue, yet current screening rarely captures such effects regionally. This exploratory study aims to quantify field cancerization via lobar-level radiomic clustering and assess its association with lung cancer risk in high-risk smokers.

**Materials and methods:**

A total of 10,280 male current or former smokers (mean age, 62.1 ± 6.34 years) were enrolled from a high-risk population undergoing lung cancer screening. Unsupervised clustering of CT-derived radiomic features was performed for each lobe. A logistic regression-derived weighted spatial risk score was developed to quantify cumulative lobar risk. Cancer incidence associations were assessed after adjusting for polygenic risk and epidemiological factors, with stratified analyses by genetic risk and smoking duration.

**Results:**

Distinct radiomic clusters were observed across all lobes, with the right upper lobe demonstrating a significantly higher lung cancer incidence in the high-risk cluster (0.997% vs 0.593%, *p* = 0.020). The weighted spatial risk score was independently associated with cancer risk (OR = 1.09, 95% CI: 1.02–1.16, *p* = 0.010). There was no significant interaction between the score and PRS (*p* = 0.938), suggesting a genetic background-independent effect. In stratified analysis, the score was significantly associated with lung cancer among long-term smokers (OR = 1.08, 95% CI: 1.00–1.17, *p* = 0.049), with a similar but nonsignificant trend in short-term smokers.

**Conclusion:**

Unsupervised clustering of lobar radiomics reveals background pulmonary alterations, supporting field cancerization and the “seed-and-soil” hypothesis, and offers an exploratory imaging-based framework for cancer risk stratification in screening.

**Critical relevance statement:**

Unsupervised clustering of radiomic features at the pulmonary lobe level identified distinct background patterns associated with nodule and cancer incidence. A derived weighted lobe score was independently associated with lung cancer risk, especially among individuals with prolonged smoking histories.

**Key Points:**

- Lobar radiomic clustering identifies background lung changes related to cancer risk.
- Weighted lobe spatial risk score predicts lung cancer risk independently of genetic background.
- Longer smoking duration strengthens the link between lung damage and cancer risk.

**Graphical Abstract:**

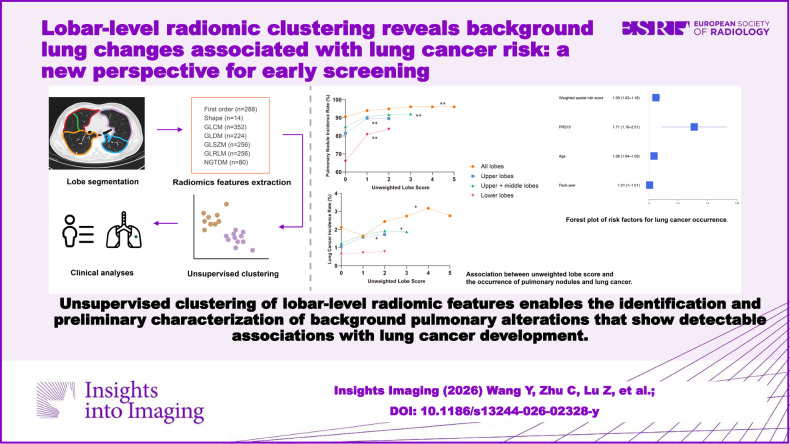

## Introduction

Lung cancer remains the leading cause of cancer-related deaths worldwide, and early detection is critical for improving prognosis [1]. Beyond tumor characteristics, increasing evidence highlights the pivotal role of the pulmonary microenvironment—particularly cumulative structural damage and tissue heterogeneity—in tumor initiation and progression [2–5]. This aligns with the concept of field cancerization, which proposes that widespread molecular and structural alterations across carcinogen-exposed lung tissue create a “preconditioned” field conducive to malignant transformation [6, 7]. For example, air pollutants can induce chronic inflammation, triggering macrophage infiltration and IL-1β release that promote malignant transformation of cells with oncogenic mutations [8]. These insights reinforce the “seed and soil” hypothesis, underscoring the importance of background lung changes in shaping cancer risk.

Imaging-based analyses have recently enabled the noninvasive characterization of such field effects, revealing subtle abnormalities in non-tumorous lung tissue that may precede visible malignancy. Emphysema exemplifies such background alterations, characterized by chronic inflammation, oxidative stress, and parenchymal destruction [9, 10]. It shares susceptibility factors and pathogenic pathways with lung cancer, and both visual and quantitative computed tomography (CT) assessments of emphysema have been associated with increased cancer risk, rising with disease severity [11]. A related study demonstrated that nodules arising in lungs with high emphysema burden but low fibrosis are more likely to be malignant, suggesting that background lung changes may help distinguish benign from malignant nodules [12].

Previous studies have primarily focused on localized lung nodules or whole-lung radiomic features, while the assessment of background tissue at the lobar level remains largely unexplored [13–16]. However, differences in ventilation, perfusion, and susceptibility to environmental exposures across pulmonary lobes may contribute to regional heterogeneity in disease progression and cancer risk [17–20]. Our previous research revealed inter-lobar differences in background features within individuals, indicating that lung aging is not a homogeneous process but exhibits distinct lobe-specific patterns through radiomics [21]. Zhao et al demonstrated that combining radiomic features from all five lobes improved classification of chronic obstructive pulmonary disease (COPD) severity, suggesting that lobe-specific analysis captures additional information beyond whole-lung assessments [22].

Carcinogenesis can be triggered through multiple pathways, while smoking-related lung cancer exhibits distinct biological behaviors and etiologies compared to non-smoking-related cases [23–26]. To reduce heterogeneity stemming from divergent carcinogenic mechanisms, we focused on smoking-induced alterations in the pulmonary background as a representative model. Specifically, we aimed to investigate whether lobar-level radiomic heterogeneity—which captures diffuse, sub-visual parenchymal injury—could provide imaging-based evidence supporting the field cancerization hypothesis in smokers.

In this exploratory study, we applied unsupervised clustering of lobar-level radiomic features to identify distinct background lung patterns [27, 28]. We further constructed a weighted spatial risk score to quantify cumulative risk and assess its association with lung cancer occurrence. We also examined its interaction with genetic susceptibility and its relevance across varying durations of smoking exposure. Rather than developing a diagnostic model, our aim was to explore and preliminarily quantify lobe-specific risk differences and evaluate their link to lung cancer.

## Methods

### Study design and data collection

Our data comes from The Wenling Lung Cancer Screening Project: Integrating Artificial Intelligence (AI) and Serum Protein Biomarkers (WELLSPING). The Wenling Lung Cancer Screening Project is part of the lung imaging genomics initiative (LIGI) in China, Phase I. The ethical board at Taizhou Cancer Hospital approved the study (IRB-2019-2). Written informed consent was obtained from all individual participants included in the study.

**Fig. 1:**
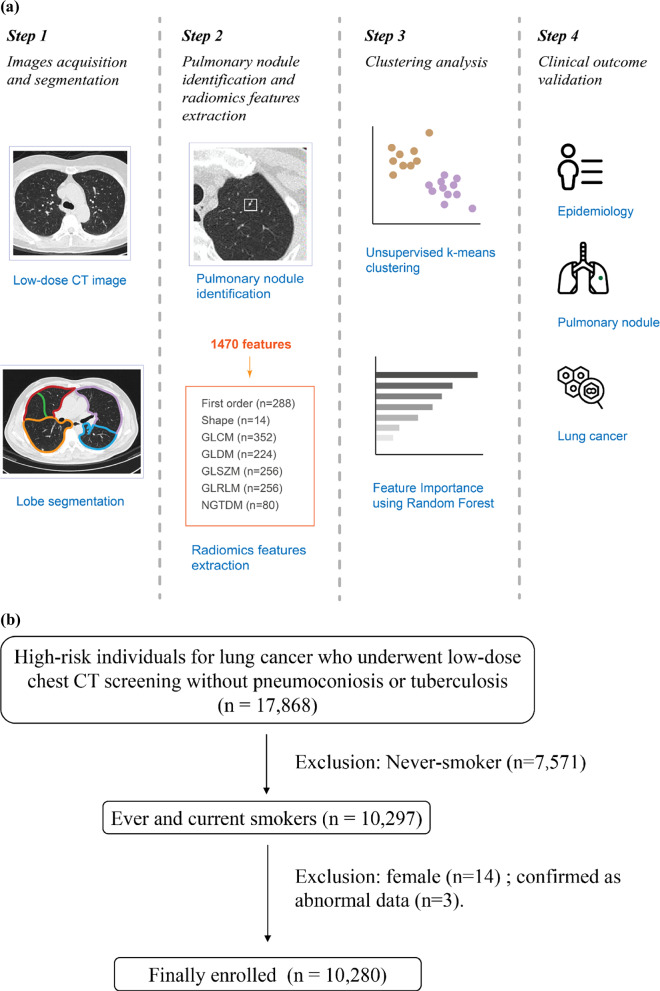
**a** Study workflow. **b** The inclusion and exclusion flowchart

The overall study process is illustrated in Fig. 1. The inclusion criteria were established according to the Lung Cancer Screening Technical Protocol of Zhejiang Province, China. Between 2019 and 2020, high-risk individuals of lung cancer were recruited from the project and subsequently underwent low-dose chest CT screening. The specific criteria were detailed in the Supplementary Material 1.

This study included current or former smokers from a cohort of individuals at high risk for lung cancer who underwent low-dose chest CT screening, excluding those diagnosed with pneumoconiosis or tuberculosis. Due to the significantly higher smoking prevalence among Chinese males (74.3%) compared to females (3.2%) [29, 30], the analysis was restricted to male participants to ensure sufficient statistical power and reliable results.

Outliers for each pulmonary lobe were defined as values below the 25th percentile minus 1.5× interquartile range (IQR) or above the 75th percentile plus 1.5× IQR of voxel volume (mm³) and CT mean (HU). Among 1283 lobes from 800 participants, two chest radiologists (each with over 8 years’ experience) independently reviewed the outliers to assess for segmentation errors or other causes. Three cases, due to segmentation failure or post-surgical changes, were excluded from further analysis. A total of 10,280 male current or former smokers (mean age, 62.1 ± 6.34 years) were finally included.

Subsequently, annual follow-ups were conducted to monitor participants’ survival outcomes. Lung cancer incidence was identified based on diagnoses from baseline screening to the end of follow-up (December 2023). Tumor location by lobe was confirmed through pathology from biopsy or surgery. Lung cancer cases in this cohort included both tumors identified at baseline and detected during follow-up. For cases with multiple lesions or with a single lesion spanning multiple lobes, the tumor location was classified as uncertain. If lesion location data were unavailable, the location was recorded as missing.

### Imaging acquisition and radiomics features extraction

Low-dose chest CT scans were acquired using a 16-row spiral scanner (120 kV, 40–60 mA; collimation 16 × 0.6 mm; pitch 1.5; 1 mm slice thickness; field of view 500 mm). Participants followed standardized breath-hold instructions during scanning (5–8 s). All analyses used baseline images from the initial screening.

We used a deep-learning AI system (Beijing Deepwise & League of PhD Technology Co., Ltd., CFDA-approved) [31] for automated segmentation of the five pulmonary lobes and lung nodules, and Pyradiomics (v3.0) for radiomic feature extraction [32]. The left lung was divided into upper (UL) and lower lobes (LL), and the right into upper (UR), middle (CR), and lower (LR) lobes. All preoperative follow-up CT images were processed using the AI system under a lung window setting (level: –600 HU, width: 1600 HU) [33].

CT-based Radiomic features were extracted using Pyradiomics (v3.0, Linux), yielding 1470 quantitative features per lobe. The original radiomics features comprised 18 First Order Statistics, 14 Shape-based (3D), 22 gray level cooccurrence matrix (GLCM), 16 gray level run length matrix (GLRLM), 16 gray level size zone matrix (GLSZM), 14 gray level dependence matrix (GLDM), and 5 neighboring gray tone difference matrix (NGTDM) features, while the image type consisted of Original, Wavelet, Laplacian of Gaussian (LoG), Square, Square Root, Logarithm, Exponential, Gradient, and LocalBinaryPattern2D.

### Genotyping and polygenic risk score (PRS) calculation

At baseline, peripheral blood samples were collected from all participants for genomic DNA extraction and genotyping. PRS was constructed based on 19 lung cancer-associated single-nucleotide polymorphisms (SNPs) identified from the genome-wide association study ‌(GWAS) results of Dai et al [34] in Chinese populations. To date, this represents the largest and most predictive PRS study conducted in Chinese cohorts. The weight (lnOR) for each SNP was derived from the fixed-effects meta-analysis results of the original study. The PRS was calculated as the weighted sum of the risk allele counts multiplied by their corresponding effect sizes. PRS19 was included as a continuous variable in further models. The detailed procedure is provided in the Supplementary Material 1.

### Clustering and statistical analysis

Unsupervised k-means clustering was performed to identify homogeneous imaging-based patient clusters in each pulmonary lobe. Silhouette analysis was used to identify the optimal number of clusters. We assigned 0 or 1 for each lobe based on clustering results. The unweighted lobe score was the sum of the scores of 5 lobes, ranging from 0 to 5 points. In light of the differential contributions of individual lung lobes to the background lung environment, a weighted standardized lobe score was derived in an exploratory manner using the β coefficients from the logistic regression model. Each lobe variable was multiplied by the β coefficients, summed, divided by the sum of the β coefficients, and multiplied by 5 [35].

$${{{\rm{Weighted}}}}\; {{{\rm{spatial}}}}\; {{{\rm{risk}}}}\; {{{\rm{score}}}}=\frac{\Sigma ({{{{\rm{\beta }}}}}_{{{{\rm{i}}}}}\times {{{{\rm{x}}}}}_{{{{\rm{i}}}}})}{\sum |{{{{\rm{\beta }}}}}_{{{{\rm{i}}}}}|}\times 5$$

Logistic regression was used to evaluate lung cancer risk and predictors. To assess potential effect modification by genetic susceptibility and smoking history, participants were stratified by median PRS and smoking duration into high/low and long/short groups, respectively. An interaction term between the weighted spatial risk score and PRS group was added to the model to test for heterogeneity. Multivariable logistic models were then constructed.

Categorical variables were compared using the χ² test, and continuous variables using Student’s *t*-test or Mann–Whitney *U*-test. Trend tests assessed associations between unweighted lobe scores and the occurrence of pulmonary nodules or lung cancer. Only one global test was performed across the five lobes (no pairwise comparisons), and all other subgroup analyses were single two-group comparisons. Thus, the number of statistical tests was limited, and no adjustment for multiple testing was applied, consistent with the exploratory nature of this study. Analyses were performed using SPSS software (version 26, SPSS for Windows, IBM, Armonk, NY, USA) and R software (version 4.3.3, R Foundation for Statistical Computing, Vienna, Austria), with two-sided *p*  <  0.05 considered significant.

## Results

### Patient characteristics

Significant differences in morphology and low-level radiomic feature distributions were observed among the five pulmonary lobes (*p* < 0.01; Supplemental eFig. S1). Each lobe was independently clustered into two subgroups using k-means clustering, guided by the silhouette coefficient (Supplemental eFig. S2). Baseline clinical characteristics corresponding to the subgroups of each pulmonary lobe are summarized in Table 1.

**Table 1:** Patient characteristics

		Right upper lobe			Right middle lobe			Right lower lobe		
	Total (*n* = 10,280)	Cluster 1 (*n* = 4716)	Cluster 2 (*n* = 5564)	*p*	Cluster 1 (*n* = 4668)	Cluster 2 (*n* = 5612)	*p*	Cluster 1 (*n* = 5045)	Cluster 2 (*n* = 5235)	*p*
Age	62.1 ± 6.34	61.7 ± 6.32	62.5 ± 6.34	< 0.01	61.8 ± 6.36	62.4 ± 6.32	< 0.01	61.6 ± 6.33	62.6 ± 6.32	< 0.01
Height	167 ± 5.80	168 ± 5.67	167 ± 5.81	< 0.01	168 ± 5.67	166 ± 5.80	< 0.01	168 ± 5.71	167 ± 5.82	< 0.01
Weight	66.2 ± 9.89	64.9 ± 9.82	67.4 ± 9.80	< 0.01	65.9 ± 10.3	66.5 ± 9.55	< 0.01	64.3 ± 9.72	68.1 ± 9.70	< 0.01
BMI	23.6 ± 3.09	22.9 ± 2.97	24.3 ± 3.05	< 0.01	23.2 ± 3.16	24.0 ± 2.99	< 0.01	22.8 ± 2.99	24.5 ± 2.95	< 0.01
Smoke				< 0.01			0.049			< 0.01
Current smoker	8544 (83.1%)	4005 (84.9%)	4539 (81.6%)		3917 (83.9%)	4627 (82.4%)		4255 (84.3%)	4289 (81.9%)	
Former smoker	1736 (16.9%)	711 (15.1%)	1025 (18.4%)		751 (16.1%)	985 (17.6%)		790 (15.7%)	946 (18.1%)	
Pack-year				0.016			0.139			0.042
< 30	557 (5.42%)	228 (4.83%)	329 (5.91%)		236 (5.06%)	321 (5.72%)		250 (4.96%)	307 (5.86%)	
≥ 30	9723 (94.6%)	4488 (95.2%)	5235 (94.1%)		4432 (94.9%)	5291 (94.3%)		4795 (95.0%)	4928 (94.1%)	
Family history	702 (6.83%)	355 (7.53%)	347 (6.24%)	0.011	358 (7.67%)	344 (6.13%)	< 0.01	387 (7.67%)	315 (6.02%)	< 0.01
Cancer history	185 (1.80%)	75 (1.59%)	110 (1.98%)	0.163	84 (1.80%)	101 (1.80%)	0.811	88 (1.74%)	97 (1.85%)	0.734
Chronic respiratory disease	751 (7.31%)	429 (9.10%)	322 (5.79%)	< 0.01	458 (9.81%)	293 (5.22%)	< 0.01	457 (9.06%)	294 (5.62%)	< 0.01
Hypertension	2823 (27.5%)	1163 (24.7%)	1660 (29.8%)	< 0.01	1196 (25.6%)	1627 (29.0%)	< 0.01	1210 (24.0%)	1618 (30.8%)	< 0.01
Hyperlipemia	808 (7.86%)	341 (7.23%)	467 (8.39%)	0.032	374 (8.01%)	434 (7.73%)	0.627	352 (6.98%)	456 (8.71%)	< 0.01
Diabetes	1170 (11.4%)	483 (10.2%)	687 (12.3%)	< 0.01	502 (10.8%)	668 (11.9%)	0.073	513 (10.2%)	657 (12.6%)	< 0.01
Nodule	9642 (93.8%)	3719 (78.9%)	3894 (70.0%)	< 0.01	2224 (47.6%)	2342 (41.7%)	< 0.01	3376 (66.9%)	2529 (48.3%)	< 0.01
Lung cancer	252 (2.45%)	47 (0.997%)	33 (0.593%)	0.020	10 (0.214%)	11 (0.196%)	0.839	18 (0.357%)	19 (0.363%)	0.958

		Left upper lobe			Left lower lobe		
	Total (*n* = 10,280)	Cluster 1 (*n* = 4838)	Cluster 2 (*n* = 5442)	*p*	Cluster 1 (*n* = 4849)	Cluster 2 (*n* = 5431)	*p*
Age, y	62.1 ± 6.34	61.6 ± 6.36	62.5 ± 6.30	< 0.01	61.6 ± 6.35	62.6 ± 6.30	< 0.01
Height, cm	167 ± 5.80	168 ± 5.63	166 ± 5.79	< 0.01	168 ± 5.73	167 ± 5.80	< 0.01
Weight, kg	66.2 ± 9.89	65.0 ± 9.92	67.3 ± 9.74	< 0.01	64.3 ± 9.89	68.0 ± 9.59	< 0.01
BMI	23.6 ± 3.09	22.9 ± 3.01	24.3 ± 3.01	< 0.01	22.8 ± 3.03	24.4 ± 2.92	< 0.01
Smoke				< 0.01			< 0.01
Current smoker	8544 (83.1%)	4120 (85.2%)	4424 (81.3%)		4127 (85.1%)	4417 (81.3%)	
Former smoker	1736 (16.9%)	718 (14.8%)	1018 (18.7%)		722 (14.9%)	1014 (18.7%)	
Pack-year				< 0.01			0.085
< 30	557 (5.42%)	231 (4.77%)	326 (5.99%)		243 (5.01%)	314 (5.78%)	
≥ 30	9723 (94.6%)	4607 (95.2%)	5116 (94.0%)		4606 (95.0%)	5117 (94.2%)	
Family history	702 (6.83%)	369 (7.63%)	333 (6.12%)	< 0.01	386 (7.96%)	316 (5.82%)	< 0.01
Cancer history	185 (1.80%)	76 (1.57%)	109 (2.00%)	0.116	85 (1.75%)	100 (1.84%)	0.793
Chronic respiratory disease	751 (7.31%)	461 (9.53%)	290 (5.33%)	< 0.01	467 (9.63%)	284 (5.23%)	< 0.01
Hypertension	2823 (27.5%)	1156 (23.9%)	1667 (30.6%)	< 0.01	1147 (23.7%)	1676 (30.9%)	< 0.01
Hyperlipemia	808 (7.86%)	340 (7.03%)	468 (8.60%)	< 0.01	330 (6.81%)	478 (8.80%)	< 0.01
Diabetes	1170 (11.4%)	491 (10.1%)	679 (12.5%)	< 0.01	496 (10.2%)	674 (12.4%)	< 0.01
Pulmonary nodule	9642 (93.8%)	3508 (72.5%)	3352 (61.6%)	< 0.01	3213 (66.3%)	2657 (48.9%)	< 0.01
Lung cancer	252 (2.45%)	37 (0.765%)	27 (0.496%)	0.084	19 (0.392%)	17 (0.313%)	0.499

Variables are expressed as mean and SD for continuous normally distributed variables, median and IQR (25th to 75th percentile) for continuous nonnormally distributed variables, and percentages for categorical variables. Chronic respiratory disease, hypertension, hyperlipemia, and diabetes are all self-reported. Pulmonary nodules/lung cancer represents the number of occurrences of pulmonary nodules/lung cancer within individuals or within each lobe. Location information of 14 cases of lung cancer is missing

Across the two subgroups identified within each pulmonary lobe, Cluster 1 consistently demonstrated higher incidence rates of pulmonary nodules compared to Cluster 2 (UR: 78.9% vs 70.0%, CR: 47.6% vs 41.7%, LR: 66.9% vs 48.3%, UL: 72.5% vs 61.6%, LL: 66.3% vs 48.9%, all have *p* < 0.01). In the UR, Cluster 1 also exhibited a significantly higher incidence of lung cancer than Cluster 2 (0.997% vs 0.593%, *p* = 0.020), whereas no statistically significant differences in lung cancer incidence were observed between subgroups in the other lobes.

Several epidemiological characteristics also differed between subgroups across all lobes (Table 1). For instance, Cluster 1 was associated with a higher prevalence of chronic respiratory disease, including COPD, emphysema, chronic bronchitis, and asthma, compared to Cluster 2 (UR: 9.10% vs 5.79%, *p* < 0.01; CR 9.81% vs 5.22%, *p* < 0.01; LR: 9.06% vs 5.62%, *p* < 0.01; UL: 9.53% vs 5.33%, *p* < 0.01; LL: 9.63% vs 5.23%, *p* < 0.01).

**Fig. 2:**
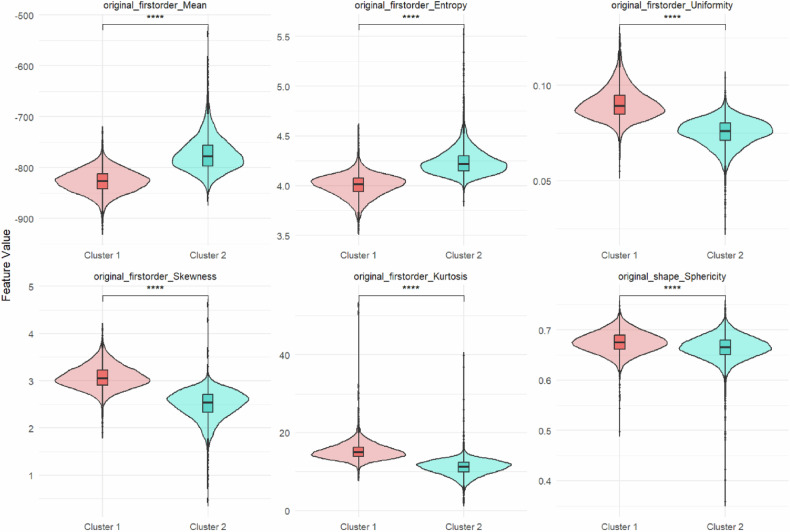
Comparison of several low-order radiomic features between clustering subgroups of the right upper lobe (using selected features as examples). ^****^p values < 0.0001

Taking several low-order features as examples, the two clustering subgroups across all five lung lobes exhibited consistent trends. Specifically, Cluster 1 was characterized by lower density and entropy, as well as higher values of uniformity, skewness, kurtosis, and relatively higher sphericity (Fig. 2 and Supplemental eFig. S3).

The overlap of clustering results across pulmonary lobes within individual participants is presented in the Supplemental eFig. S4.

### Cluster analysis

**Fig. 3:**
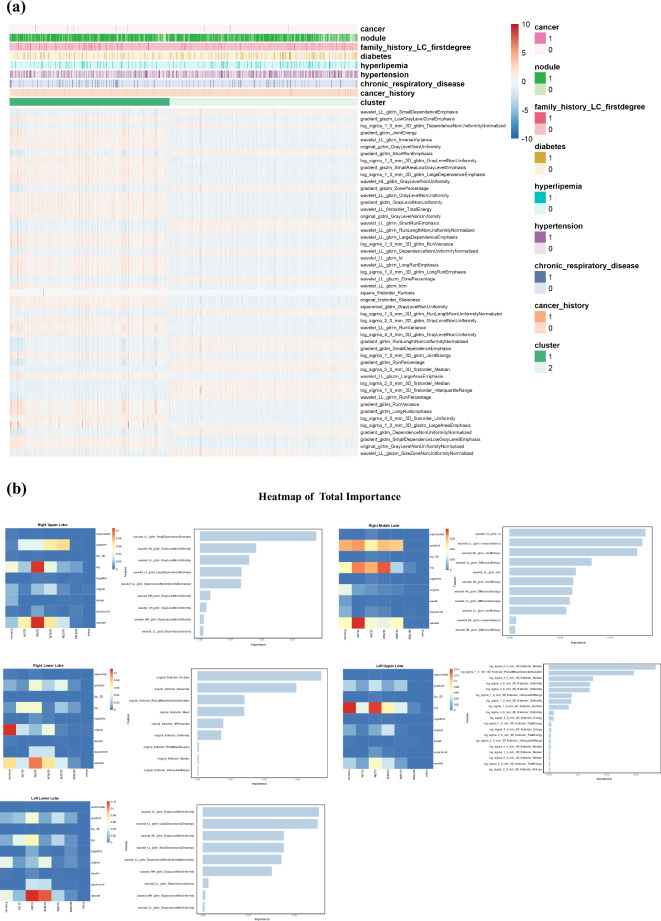
Clustering analysis. **a** Cluster heatmap (top 50 contributing features selected from the clustering, example: right upper lobe). **b** Feature contribution analysis for each pulmonary lobe cluster (top 200 features). Left: heatmap summarizing contributions by feature category. Right: bar chart showing detailed distribution within the category with the highest total contribution

The heatmap in Fig. 3a (illustrated using the right upper lobe as an example) presents the results of unsupervised k-means clustering, highlighting the distribution of the top 50 most influential radiomic features differentiating the two subgroups. Other lobes are shown in the Supplemental eFig. S5.

Figure 3b summarizes the types of radiomic features among the top 200 clustering-contributing features, along with their corresponding image transformations for each pulmonary lobe. Among the transformation methods, LoG, Wavelet, and Gradient filters contributed substantially to clustering performance. The associated texture features derived from these transformations played a critical role in distinguishing subgroups. Notably, the right lower lobe (LR) included a top-ranked feature derived from original first-order statistics, indicating a unique contribution pattern.

### Unweighted lobe score and analysis

**Fig. 4:**
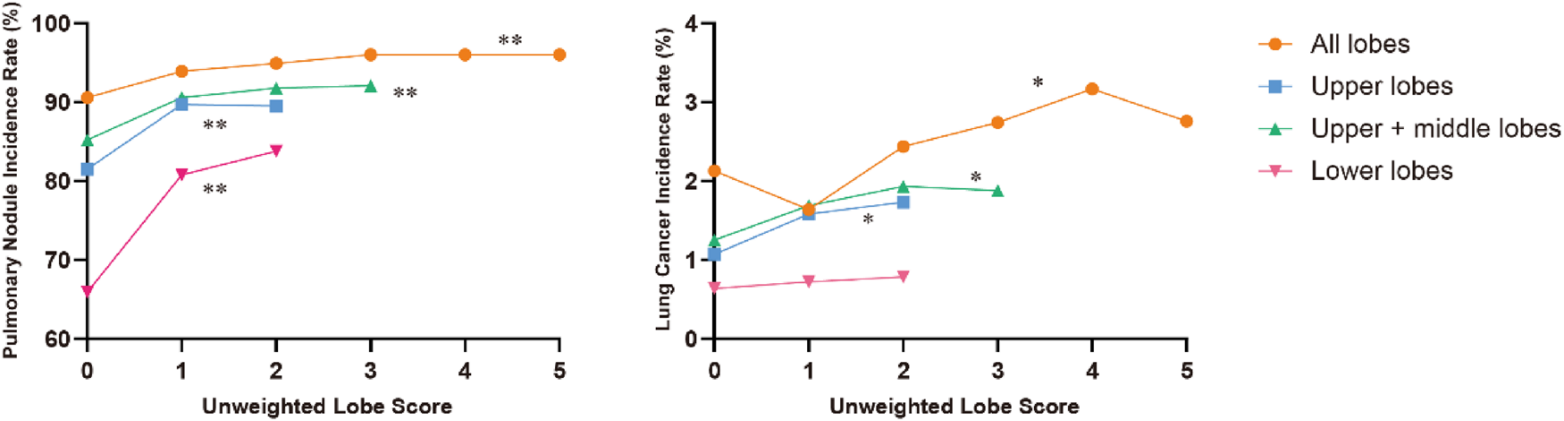
Association between unweighted lobe score and the occurrence of pulmonary nodules and lung cancer. Trend analysis across different lobe score groups. Note: based on the trend test, ^*^p values < 0.05, ^**^p values < 0.01

Based on the distribution of radiomic features, relevant clinical indicators, and most notably the incidence of pulmonary nodules, Cluster 1 was assigned a score of 1 (high risk) and Cluster 2 a score of 0 (low risk). The unweighted lobe score, ranging from 0 to 5, was calculated by summing the scores across the five lung lobes for each individual. As the unweighted lobe score increased, the incidence of pulmonary nodules showed an upward trend (90.6%–93.9%–94.9%–96.0%–96.0%–96.0%; *p* for trend < 0.01). At the same time, the incidence of lung cancer also demonstrated a fluctuating but overall upward pattern (2.13%–1.64%–2.44%–2.74%–3.17%–2.76%; *p* for trend < 0.01) (Table 2 and Fig. 4).

**Table 2:** Pulmonary nodules and lung cancer occurrence across groups with different unweighted lobe scores

(a) All lobes							
Unweighted lobe score	0 (*n* = 3617)	1 (*n* = 973)	2 (*n* = 818)	3 (*n* = 875)	4 (*n* = 1103)	5 (*n* = 2894)	*p* for trend
Lung cancer	77 (2.13%)	16 (1.64%)	20 (2.44%)	24 (2.74%)	35 (3.17%)	80 (2.76%)	0.020
Pulmonary nodule	3276 (90.6%)	914 (93.9%)	776 (94.9%)	840 (96.0%)	1059 (96.0%)	2777 (96.0%)	< 0.01

(b) Upper lobes				
Unweighted lobe score	0 (*n* = 4773)	1 (*n* = 1460)	2 (*n* = 4047)	*p* for trend
Lung cancer	51 (1.07%)	23 (1.58%)	70 (1.73%)	0.008
Pulmonary nodule	3888 (81.5%)	1310 (89.7%)	3623 (89.5%)	< 0.01

(c) Upper + middle lobes					
Unweighted lobe score	0 (*n* = 4228)	1 (*n* = 1241)	2 (*n* = 1452)	3 (*n* = 3359)	*p* for trend
Lung cancer	53 (1.25%)	21 (1.69%)	28 (1.93%)	63 (1.88%)	0.024
Pulmonary nodule	3601 (85.2%)	1124 (90.6%)	1333 (91.8%)	3094 (92.1%)	< 0.01

(d) Lower lobes				
Unweighted lobe score	0 (*n* = 4709)	1 (*n* = 1248)	2 (*n* = 4323)	*p* for trend
Lung cancer	30 (0.637%)	9 (0.721%)	34 (0.786%)	0.398
Pulmonary nodule	3101 (65.9%)	1008 (80.8%)	3622 (83.8%)	< 0.01

Stratified analyses by lung region further revealed that in the upper lobes, both pulmonary nodule prevalence (81.5%–89.7%–89.5%; *p* for trend < 0.01) and lung cancer incidence (1.07%–1.58%–1.73%; *p* for trend < 0.01) increased with higher unweighted lobe scores. In contrast, in the lower lobes, only pulmonary nodules exhibited a significant increasing trend (65.9%–80.8%–83.8%; *p* for trend < 0.01), while lung cancer incidence showed no significant trend (0.637%–0.721%–0.786%; *p* for trend = 0.398). These findings suggest that the contribution of lobar clustering subgroups to the overall pathological background may vary by anatomical location within the lung.

### Construction of a weighted spatial risk score and a regression model

Using logistic regression modeling, coefficients for each pulmonary lobe were adjusted to generate a weighted spatial risk score (Supplemental eTable S1). After adjustment for genetic and epidemiological factors, only significant variables were retained in the final model, as shown in Table 3(a).

**Table 3:** Logistic regression model

(a) Multivariable logistic regression analysis for lung cancer risk in the overall population		
Variable	OR (95% CI)	*p*
Weighted spatial risk score	1.09 (1.02–1.16)	0.010
PRS19	1.71 (1.16–2.51)	0.0066
Age	1.06 (1.04–1.09)	< 0.001
Pack_year	1.01 (1.00–1.01)	0.005

(b) Multivariable logistic regression models for stratified and interaction analysis between the weighted spatial risk score and PRS groups		
Variable	OR (95% CI)	*p*
Weighted spatial risk score	1.08 (0.99, 1.18)	0.068
PRS_group (low vs high)	0.82 (0.54, 1.25)	0.359
Age	1.06 (1.04, 1.09)	< 0.001
Pack_year	1.01 (1.00, 1.01)	0.006
Interaction: weighted spatial risk score × PRS_Low	1.01 (0.89, 1.14)	0.938

(c) Multivariable logistic regression analysis of lung cancer risk stratified by years of smoking			
Smoking duration group	Variable	OR (95% CI)	*p*
Long-term smokers	Weighted spatial risk score	1.08 (1.00–1.17)	0.049
	PRS19	1.78 (1.10–2.85)	0.0175
	Age	1.06 (1.02–1.10)	0.0034
Short-term smokers	Weighted spatial risk score	1.09 (0.98–1.21)	0.131
	PRS19	1.55 (0.80–3.00)	0.193
	Age	1.07 (1.03–1.11)	0.001

*OR* odds ratio, *CI* confidence interval, *PRS* polygenic risk score

**Fig. 5:**
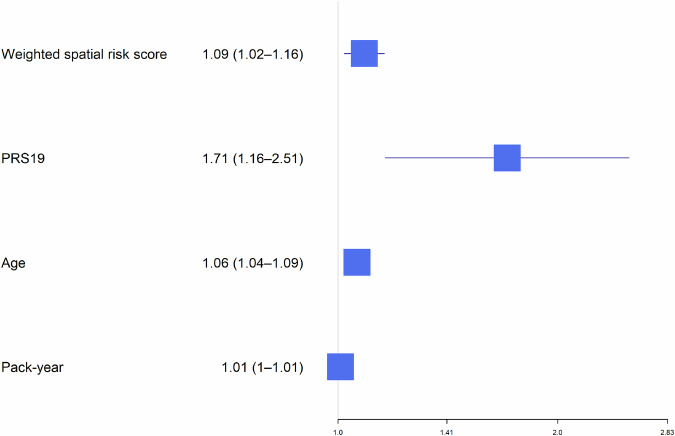
Forest plot of risk factors for lung cancer occurrence. Forest plot displaying the associations between key variables and lung cancer risk based on a multivariate logistic regression model in the overall population. The weighted spatial risk score, a structural imaging-based index, showed a significant association with cancer risk. Each line represents the 95% confidence interval, with the square indicating the odds ratio (OR), and the vertical dashed line marking the reference (OR = 1)

The weighted spatial risk score showed an independent, monotonic association with cancer risk (per-unit OR = 1.09, 95% CI: 1.02–1.16, *p* = 0.010). PRS19 (OR = 1.71, 95% CI: 1.16–2.51, *p* < 0.01), age (OR = 1.06, 95% CI: 1.04–1.09, *p* < 0.01), and cumulative smoking exposure (pack-year) (OR = 1.01, 95% CI: 1.00–1.01, *p* < 0.01) were also significantly associated with lung cancer. Figure 5 forest plot illustrates effect sizes alongside age, pack-year, and PRS19.

In the PRS interaction model (Table 3(b)), neither the weighted spatial risk score (OR = 1.08, 95% CI: 0.99–1.18, *p* = 0.068) nor the PRS group (low vs high, OR = 0.82, 95% CI: 0.54–1.25, *p* = 0.359) demonstrated a significant main effect. Interaction analysis indicated that the association between the weighted spatial risk score and lung cancer did not differ significantly between the low and high PRS groups (interaction OR = 1.01, 95% CI: 0.89–1.14, *p* = 0.938).

In the stratified analysis by smoking duration (Table 3(c)), the weighted spatial risk score was significantly associated with lung cancer risk among long-term smokers (OR = 1.08, 95% CI: 1.00–1.17, *p* = 0.049). In short-term smokers, a similar positive trend was observed, though it did not reach statistical significance (OR = 1.09, 95% CI: 0.98–1.21, *p* = 0.131). Both PRS19 (OR = 1.78, 95% CI: 1.10–2.85, *p* = 0.0175) and age (OR = 1.06, 95% CI: 1.02–1.10, *p* < 0.01) were also significant risk factors in the long-term smoking group.

## Discussion

Our work extends the classical theory of field cancerization—which posits that carcinogen-exposed tissue regions undergo widespread, pre-malignant transformation—by providing quantitative imaging-based evidence of regional field effects in the human lung. Utilizing a large screening cohort and fully automated lobar radiomic clustering, our exploratory analysis suggests that structural and microenvironmental injury is not homogeneously distributed, but may instead be regionally accentuated—most notably in the right upper lobe. This spatial heterogeneity is associated with differential cancer risk, suggesting the existence of anatomical hotspots within the carcinogen-exposed lung. For decades, lung cancer risk assessment has predominantly relied on nodule-centric models, which may overlook broader contextual factors. Our previous work [36] established that a model integrating multiple nodules and global lung features outperformed nodule-only models for long-term lung cancer risk prediction and enabled risk prediction in screening-negative participants. Extending this, we now investigate the role of lobar heterogeneity in lung cancer development. To our knowledge, this exploratory study provides the first quantitative imaging evidence that field cancerization—a spatially orchestrated, regionally accentuated risk landscape—can be preliminarily mapped and measured in the human lung. In this male smoker cohort, the weighted spatial radiomic risk score, derived from routine low-dose CT, was found to be independently associated with lung cancer occurrence at the individual level, though these findings require validation in broader populations.

Previous research has revealed that environmental factors—such as air pollution, chronic inflammation, and local tissue injury—and genetic susceptibility (PRS, for example) contribute to lung carcinogenesis through both independent and interactive pathways [4, 8, 26, 34]. In this exploratory study, we observed no significant interaction between the weighted spatial risk score and PRS, which may indicate that the spatial risk captured by lobar radiomic phenotypes reflects an acquired microenvironmental pathway that operates largely independently of inherited genetic susceptibility, with a consistent predictive direction across different genetic risk profiles. This pattern, though preliminary, highlights a potential role for localized, non-genetic injury—often overlooked in traditional risk models—and suggests that imaging-based field mapping may, after independent validation, have potential utility for risk stratification and surveillance in future studies. Our analysis also identified a stronger association between the weighted spatial risk score and lung cancer risk among long-term smokers, suggesting that cumulative tobacco exposure may exacerbate structural lung damage and heighten microenvironmental susceptibility to malignancy, with a consistent effect direction across different exposure groups. These findings, to some extent, support the concept of field cancerization, in which widespread tissue injury—driven by chronic inflammation and remodeling—primes the lung environment for carcinogenesis beyond direct mutagenic effects [3, 4, 8, 37–39].

A single low-dose CT scan enables the extraction of thousands of lobe-specific features (5 lobes × ~1000 features). Compared to purely “nodule-level” or “whole-lung-level” analyses, lobe-level resolution preserves local microstructural information while aligning with known pathological and physiological heterogeneity [11, 17, 18, 20, 40–42]. For example, lung cancer tends to predominate in the upper lobes, whereas chronic inflammation or fibrosis is more common in the lower lobes [11, 20, 41, 42]. These distributions may be rooted in inter-lobar differences in bronchial anatomy, airflow, and perfusion. A recent lung imaging phenotyping study involving 35,469 Chinese participants revealed that while heritability showed no significant differences across lung lobes, 103 genetic loci were significant in only a single lobe [43]. This finding reinforces the understanding that the lung is a highly heterogeneous organ and underscores the importance of lobe-specific analysis. Physiologically, the right upper lobe bronchus, for instance, has relatively low airflow velocity [44, 45], and ventilation–perfusion relationships vary significantly across lobes [46]. Such physiological distinctions can shape radiomic patterns by influencing local oxygenation, particle deposition, and immune activity. Lower blood flow in the upper lobes may impair carcinogen clearance, while higher perfusion in the lower lobes supports gas exchange and pathogen removal [18, 19]. This imbalance may partly explain the greater vulnerability of the upper lobes to malignant transformation and supports the rationale for lobe-specific radiomic analysis in early cancer detection.

In our exploratory analysis, the observation that only the right upper lobe showed significant differences in lung cancer incidence between radiomic clusters may reflect the current distribution of cases and the physiological distinctions described above, suggesting that upper lobe damage may be more pronounced and thus more readily captured by radiomic analysis. Significant differences in pulmonary nodule incidence were observed across clustering subgroups of each lobe, with the upper lobes showing a higher prevalence of nodules. Although this study did not stratify nodules by Lung-RADS categories, the presence of pulmonary nodules can, to some extent, be interpreted as a manifestation of underlying pathological changes, including inflammatory infiltration, fibrotic remodeling, or emphysematous destruction. With longer follow-up, we speculate that similar trends in cancer incidence may emerge across clustering subgroups of other lobes.

From the perspective of several clinically interpretable low-order radiomic features, the high-risk lobe clusters tended to exhibit lower density and higher sphericity, which may reflect emphysematous alterations. Additionally, features such as kurtosis, skewness, and uniformity were associated with background heterogeneity, potentially reflecting underlying structural and functional abnormalities within the lung parenchyma. To further capture the complexity of lung parenchymal alterations, more advanced radiomic features were examined. Radiomic features, particularly those derived from LoG, wavelet, and gradient transformations, enhance the detection of parenchymal heterogeneity by capturing both large-scale and fine structural abnormalities [47–51]. LoG emphasizes local high-contrast structures and was especially informative in upper-lobe regions, potentially reflecting emphysematous changes [11, 52]. Wavelet decomposition enables multi-scale analysis, distinguishing between coarse patterns like fibrosis and fine textures such as micronodules [53–57]. Gradient-based features highlight abrupt density transitions and disrupted anatomical organization, making them sensitive to both fibrotic distortion and alveolar destruction [58, 59]. Together, these approaches enhance characterization of diffuse background remodeling potentially relevant to lung cancer risk.

Our study has several limitations. First, clustering was performed at the lobar level, while subgroup analyses were at the individual level, potentially weakening associations with clinical factors. Second, lung cancer development requires long-term follow-up, and the relatively low incidence at the current time point may limit statistical power. Despite observing statistically significant differences within the current limited follow-up period, an extended follow-up will be conducted to enable a more comprehensive assessment. Third, as the weighted spatial risk score was both derived and internally evaluated using the same dataset, the reported performance may be subject to optimism bias. Thus, the absolute estimates should be interpreted with caution. Independent external validation is required before any clinical applications can be considered. Besides, our analysis adjusted for a core set of confounders based on data availability and prior knowledge of established lung cancer risk factors. This limited set was chosen to maintain model parsimony and to focus on the most relevant confounders in our male smoker cohort. Nevertheless, we acknowledge that unmeasured or residual confounding factors (e.g., occupational exposures, ambient air pollution, family history of lung cancer, or socioeconomic status) may still exist and could partially influence the observed associations. Moreover, our study population was limited to male smokers. Given the marked gender disparity in smoking prevalence in China and the potentially distinct mechanisms of lung carcinogenesis in non-smokers, the generalizability of our findings to females, non-smokers, or other ethnic populations requires validation in independent, diverse cohorts with an expanded set of covariates. Additionally, extending analysis to the pulmonary segment level may enable more granular investigation.

In conclusion, unsupervised clustering of lobar-level radiomic features enables the identification and preliminary characterization of background pulmonary alterations, providing imaging evidence that aligns with the field cancerization theory and the “seed-and-soil” hypothesis. This approach may offer a preliminary, exploratory framework for future investigation into lung cancer risk stratification in high-risk populations.

## Supplementary information

ELECTRONIC SUPPLEMENTARY MATERIAL

## Data Availability

The genome-wide summary statistics have been deposited in the Genome Variation Map (GVM) at the National Genomics Data Center, China National Center for Bioinformation, under accession number GVP000047 (https://ngdc.cncb.ac.cn/gvm/). Researchers may request access through the CNCB online application system by submitting a brief description of the intended scientific use. Requests are reviewed by the LIGI data access committee. Data sharing requests for anonymized imaging data, analysis scripts, or other pre-specified data will be considered by the co-authors upon reasonable written request to the corresponding author. Availability is subject to submission of a formal research proposal and a signed data sharing agreement, in accordance with data protection regulations and approval by the relevant data access committee.

## Abbreviations

AI: Artificial intelligence
COPD: Chronic obstructive pulmonary disease
CR: Right middle lobe
CT: Computed tomography
GLCM: Gray level cooccurrence matrix
GLDM: Gray level dependence matrix
GLRLM: Gray level run length matrix
GLSZM: Gray level size zone matrix
GWAS: Genome-wide association study
IQR: Interquartile range
LIGI: Lung imaging genomics initiative
LL: Left lower lobe
LoG: Laplacian of Gaussian
LR: Right lower lobe
NGTDM: Neighboring gray tone difference matrix
PRS: Polygenic risk score
SNPs: Single-nucleotide polymorphisms
UL: Left upper lobe
UR: Right upper lobe

## Code availability

A deep-learning AI system (Beijing Deepwise & League of PhD Technology Co., Ltd., CFDA-approved) was used for lung lobe segmentation and nodule extraction. This is a commercial software product; researchers who wish to use the same segmentation system may contact Deepwise Medical for commercial licensing or collaboration. Radiomic feature extraction was performed using the open-source package Pyradiomics (v3.0). The code used to perform the analyses are available here: https://github.com/terryli710/COVID_19_Rapid_Triage_Risk_Predictor.
