# Supplementary material for: Lobar-level radiomic clustering reveals background lung changes associated with lung cancer risk: a new perspective for early screening
Source: Insights Imaging. 2026 Jun 20;17:165. doi: 10.1186/s13244-026-02328-y (PMC13283237; doi:10.1186/s13244-026-02328-y)
Supplement: Supplementary file 1 — ELECTRONIC SUPPLEMENTARY MATERIAL [file 13244_2026_2328_MOESM1_ESM.pdf]

Lobar-Level Radiomic Clustering Reveals Background Lung Changes Associated with Lung Cancer Risk: A New Perspective for Early Screening

ELECTRONIC SUPPLEMENTARY MATERIAL

Supplemental material 1

Lung cancer screening technical protocol

Those who aged 50 to 74 years who meet at least one of the following criteria were defined as high-risk individuals:

(a) ≧20 pack-years of smoking a smoking, including those who have quitted smoking for less than 15 years after smoking for ≧20 pack-years;

(b) ≧20 years of passive smoking exposure from working or living with someone who meets criterion (a);

(c) diagnosis of chronic obstructive pulmonary disease (COPD);

(d) occupational exposure of at least 1 year to carcinogens such as asbestos, radon, beryllium, chromium, cadmium, silica, coal smoke, and coal ash;

(e) first-degree relatives (parents, children and siblings) have been diagnosed with lung cancer.

Genotyping and polygenic risk score (PRS) calculation

Genotyping was performed using the Illumina Asian Screening Array platform, with standard quality control procedures applied. Single Nucleotide Polymorphisms (SNPs) with a minor allele frequency (MAF) < 0.01, Hardy-Weinberg equilibrium $P < 1\times10^{-6}$, or high missing call rates were excluded.

We calculated the polygenic risk score (PRS19) based on a model developed by Dai et al. (2019)[23] for lung cancer susceptibility in Chinese populations, which includes 19 risk-associated SNPs. The PRS19 for each individual was calculated as a weighted sum of the risk alleles:

$$\mathrm{PRS19} = \sum_{i=1}^{19} \beta_i \times G_i$$

where G_i represents the number of risk alleles (0, 1, or 2) at the ith SNP, and β_i is the corresponding log odds ratio derived from the original study. The resulting PRS19 was treated as a continuous variable in regression analyses.

Supplemental eTable S1: Logistic regression coefficients for lung cancer risk of different pulmonary lobes.

Variable	β	OR (95% CI)	P
**UR**	0.176	1.19 (0.91–1.57)	0.210
**CR**	0.123	1.13 (0.86–1.48)	0.372
**LR**	0.281	1.06 (1.00-1.76)	0.051
**UL**	0.267	1.01 (0.99–1.72)	0.058
**LL**	0.247	1.09 (0.97–1.70)	0.085

Abbreviations: UR = right upper lobe; CR = right middle lobe; LR = right lower lobe; UL = left upper lobe; LL = left lower lobe.

Supplemental eFigure S1: Visualization of shape and first-order radiomic features across the five pulmonary lobes.

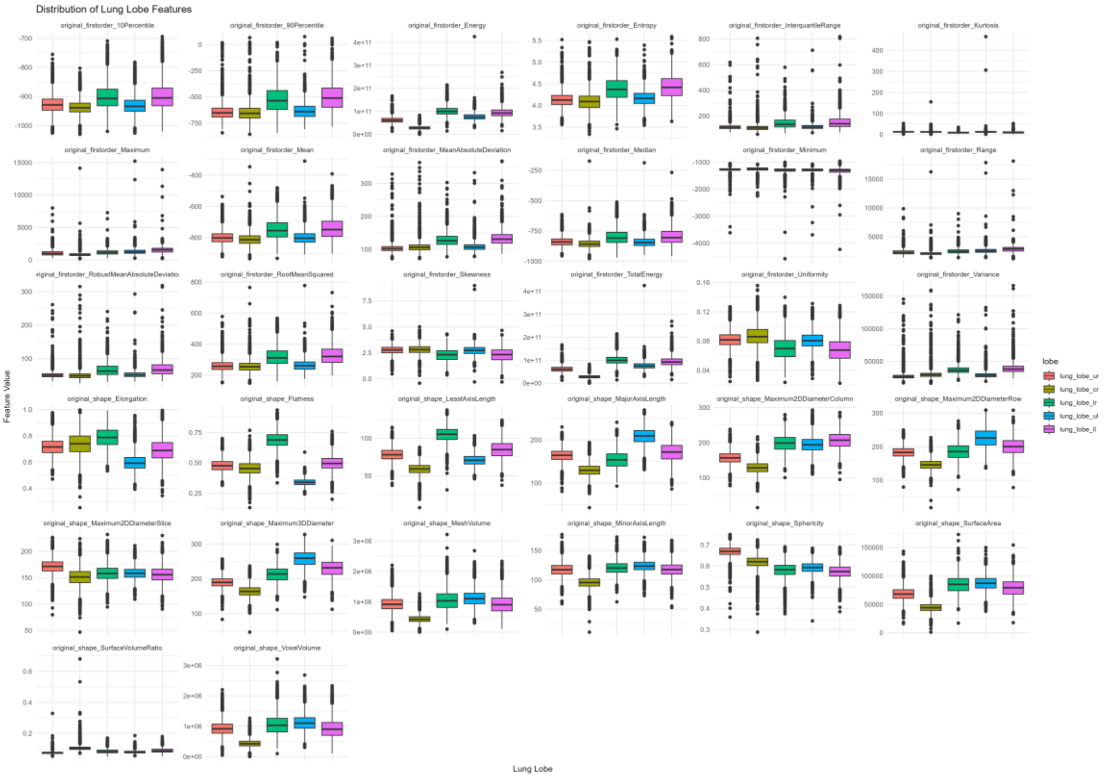

Supplemental eFigure S2: Silhouette coefficients for each pulmonary lobe cluster.

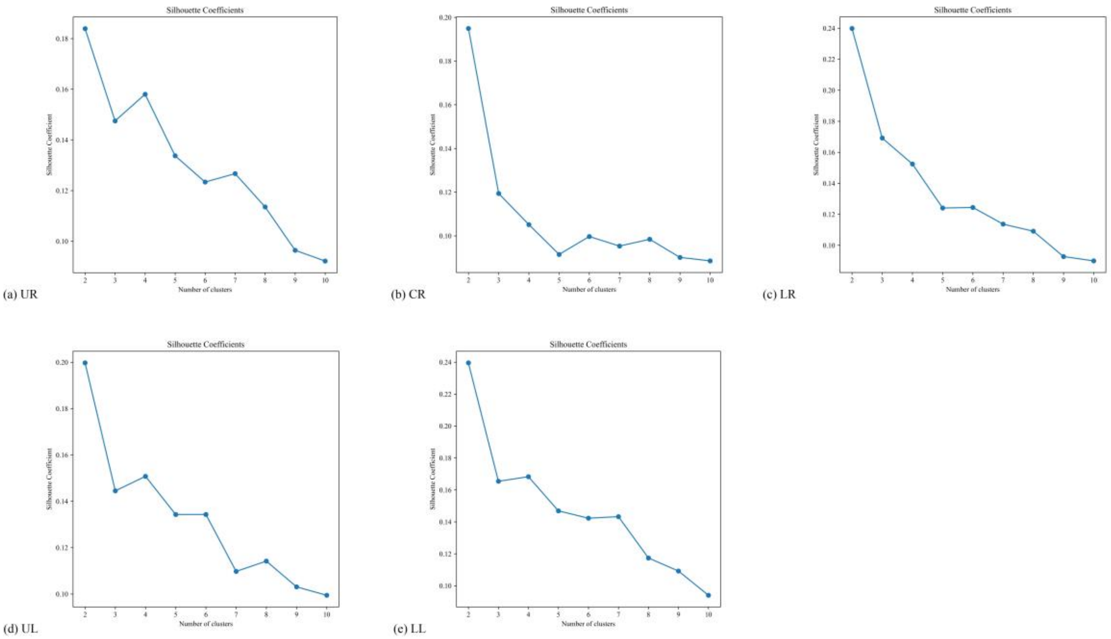

Supplemental eFigure S3: Comparison of low-order radiomic features between clustering subgroups of the other four lung lobes (using selected features as examples).

(a) CR

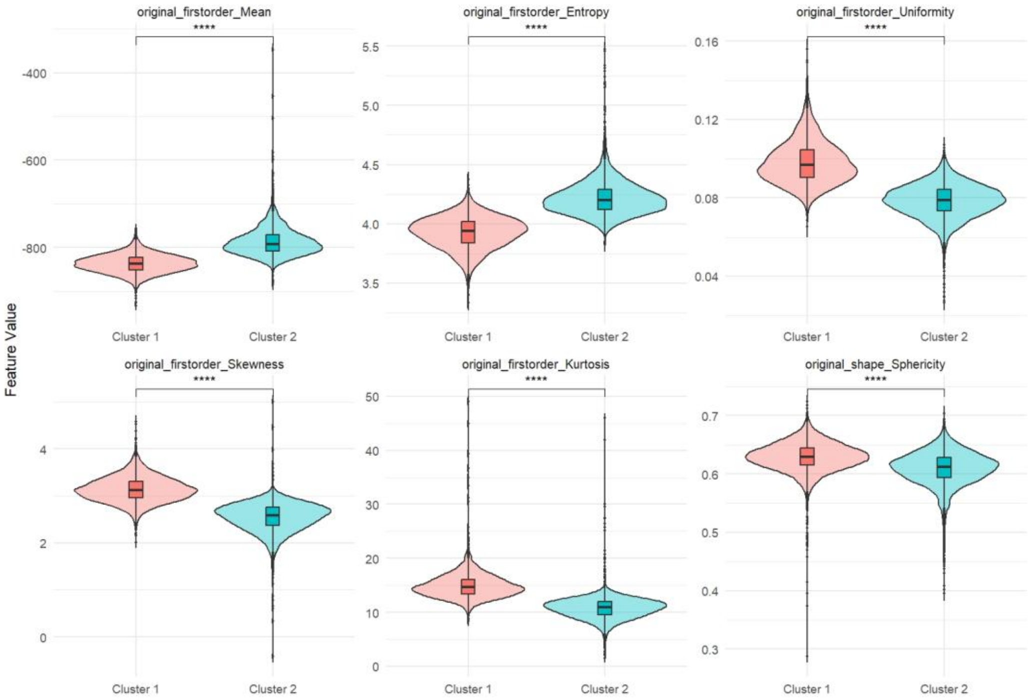

(b) LR

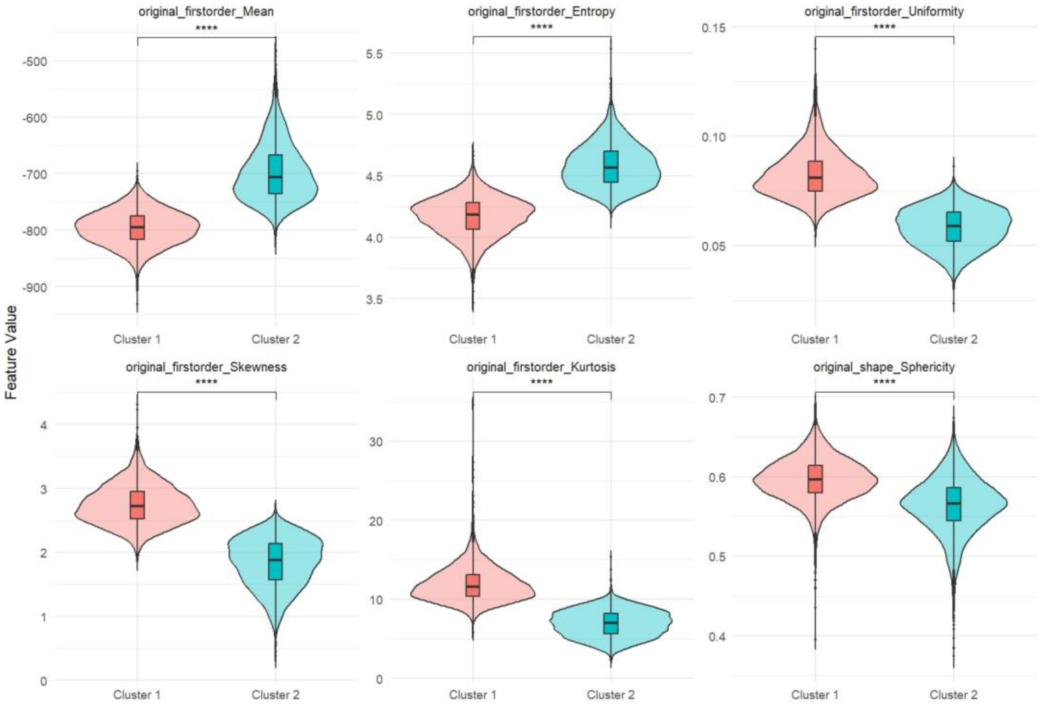

(c) UL

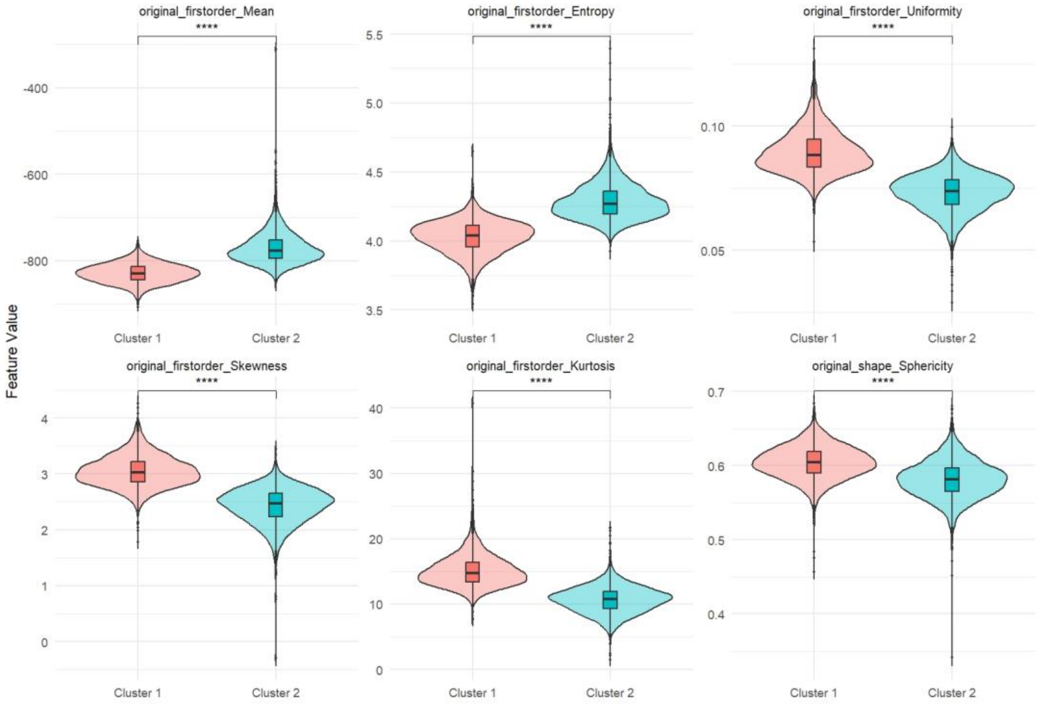

original_firstorder_Mean
original_firstorder_Entropy
original_firstorder_Uniformity
original_firstorder_Skewness
original_firstorder_Kurtosis
original_shape_Sphericity
Feature Value
Cluster 1
Cluster 2
****

(d) LL

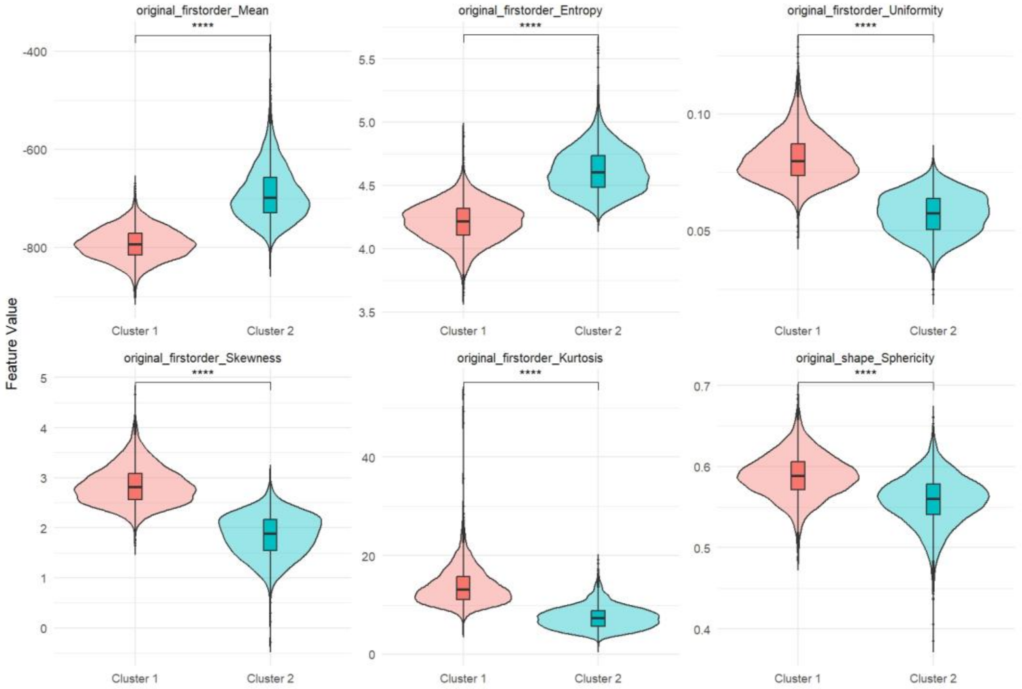

original_firstorder_Mean
original_firstorder_Entropy
original_firstorder_Uniformity
original_firstorder_Skewness
original_firstorder_Kurtosis
original_shape_Sphericity
Feature Value
Cluster 1
Cluster 2
****

Supplemental eFigure S4: The overlap among each pulmonary lobe cluster subgroups.

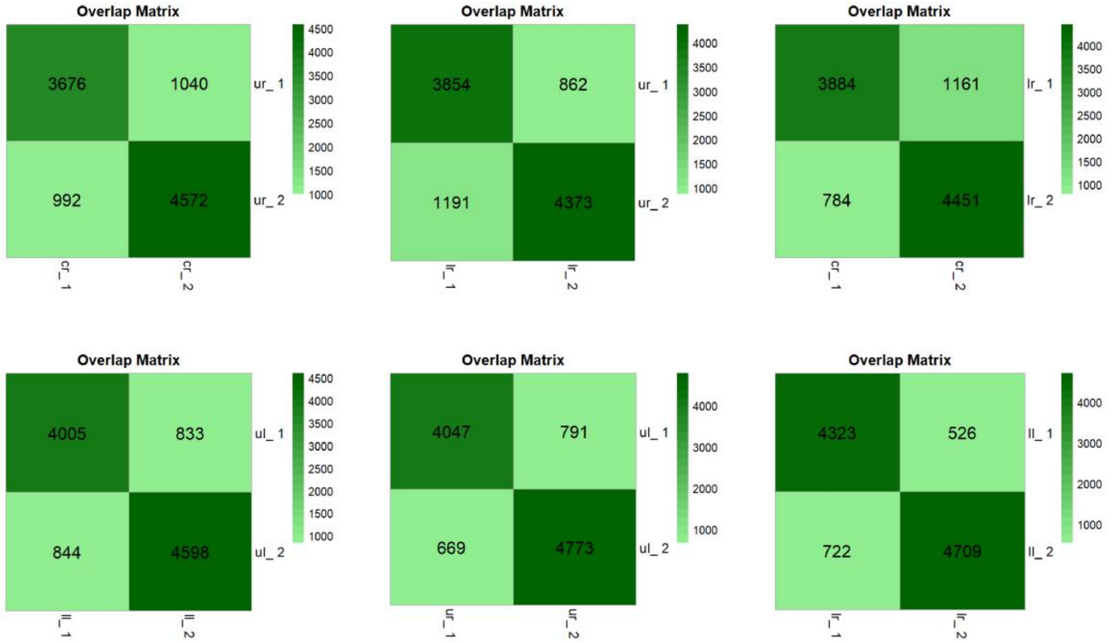

Supplemental eFigure S5: Cluster heatmaps of the other four pulmonary lobes.

(a) CR

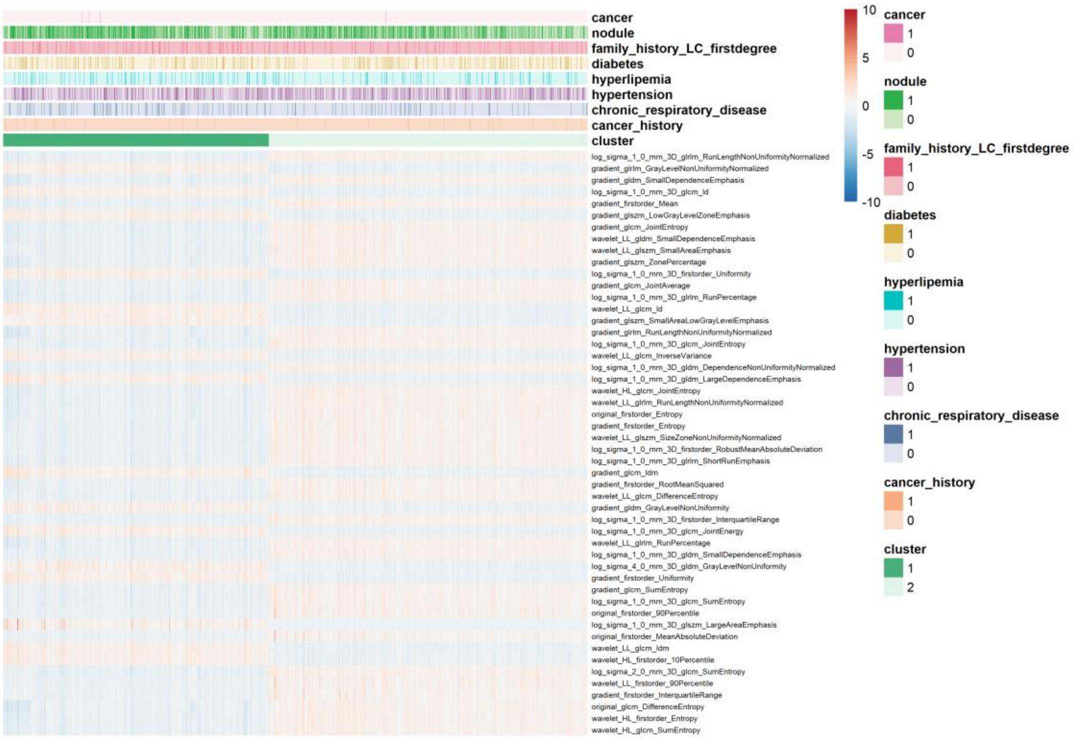

(b) LR

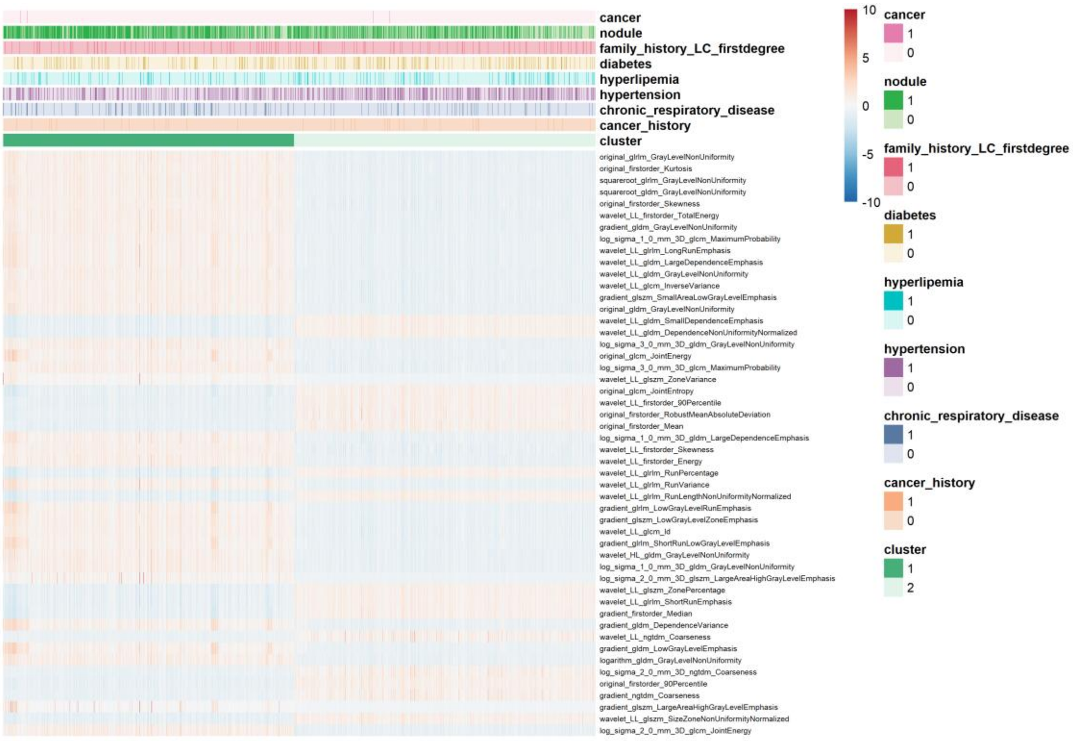

(c) UL

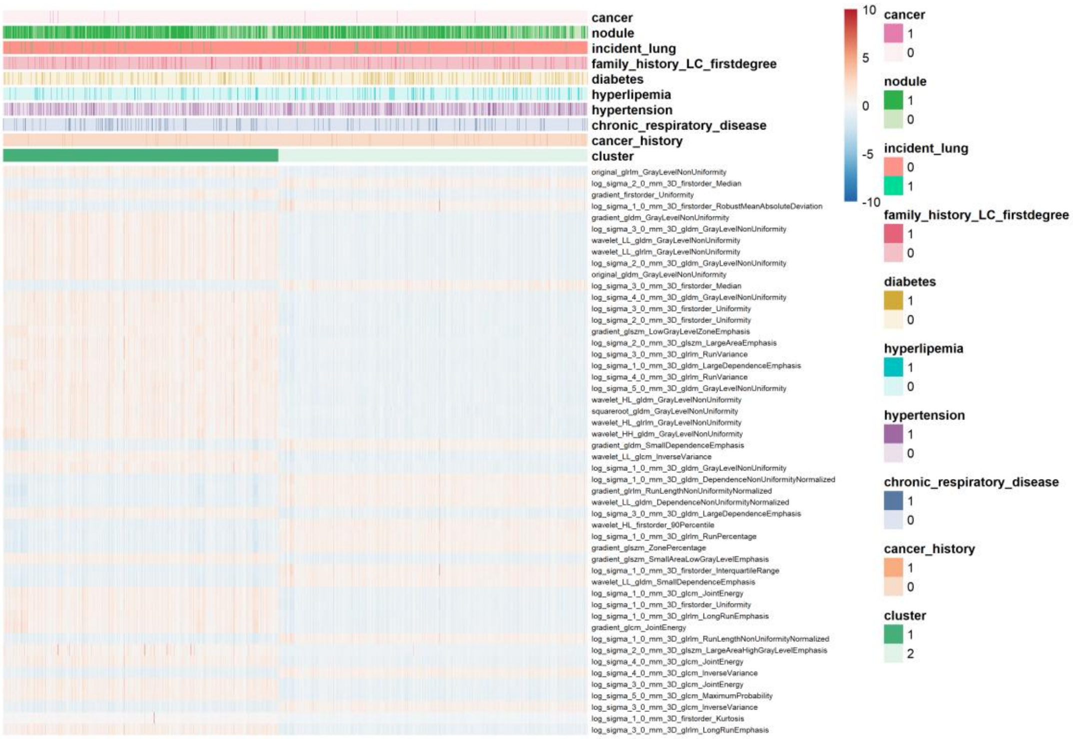
cancer
nodule
incident_lung
family_history_LC_firstdegree
diabetes
hyperlipemia
hypertension
chronic_respiratory_disease
cancer_history
cluster
original_glrlm_GrayLevelNonUniformity
log_sigma_2_0_mm_3D_firstorder_Median
gradient_firstorder_Uniformity
log_sigma_1_0_mm_3D_firstorder_RobustMeanAbsoluteDeviation
gradient_gldm_GrayLevelNonUniformity
log_sigma_3_0_mm_3D_gldm_GrayLevelNonUniformity
wavelet_LL_gldm_GrayLevelNonUniformity
wavelet_LL_glrlm_GrayLevelNonUniformity
log_sigma_2_0_mm_3D_gldm_GrayLevelNonUniformity
original_gldm_GrayLevelNonUniformity
log_sigma_3_0_mm_3D_firstorder_Median
log_sigma_4_0_mm_3D_gldm_GrayLevelNonUniformity
log_sigma_3_0_mm_3D_firstorder_Uniformity
log_sigma_2_0_mm_3D_firstorder_Uniformity
gradient_glszm_LowGrayLevelZoneEmphasis
log_sigma_2_0_mm_3D_glszm_LargeAreaEmphasis
log_sigma_3_0_mm_3D_glrlm_RunVariance
log_sigma_1_0_mm_3D_gldm_LargeDependenceEmphasis
log_sigma_4_0_mm_3D_glrlm_RunVariance
log_sigma_5_0_mm_3D_gldm_GrayLevelNonUniformity
wavelet_HL_gldm_GrayLevelNonUniformity
squareroot_gldm_GrayLevelNonUniformity
wavelet_HL_glrlm_GrayLevelNonUniformity
wavelet_HH_gldm_GrayLevelNonUniformity
gradient_gldm_SmallDependenceEmphasis
wavelet_LL_glcm_InverseVariance
log_sigma_1_0_mm_3D_gldm_GrayLevelNonUniformity
log_sigma_1_0_mm_3D_gldm_DependenceNonUniformityNormalized
gradient_glrlm_RunLengthNonUniformityNormalized
wavelet_LL_gldm_DependenceNonUniformityNormalized
log_sigma_3_0_mm_3D_gldm_LargeDependenceEmphasis
wavelet_HL_firstorder_90Percentile
log_sigma_1_0_mm_3D_glrlm_RunPercentage
gradient_glszm_ZonePercentage
gradient_glszm_SmallAreaLowGrayLevelEmphasis
log_sigma_1_0_mm_3D_firstorder_InterquartileRange
wavelet_LL_gldm_SmallDependenceEmphasis
log_sigma_1_0_mm_3D_glcm_JointEnergy
log_sigma_1_0_mm_3D_firstorder_Uniformity
log_sigma_1_0_mm_3D_glrlm_LongRunEmphasis
gradient_glcm_JointEnergy
log_sigma_1_0_mm_3D_glrlm_RunLengthNonUniformityNormalized
log_sigma_2_0_mm_3D_glszm_LargeAreaHighGrayLevelEmphasis
log_sigma_4_0_mm_3D_glcm_JointEnergy
log_sigma_4_0_mm_3D_glcm_InverseVariance
log_sigma_3_0_mm_3D_glcm_JointEnergy
log_sigma_5_0_mm_3D_glcm_MaximumProbability
log_sigma_3_0_mm_3D_glcm_InverseVariance
log_sigma_1_0_mm_3D_firstorder_Kurtosis
log_sigma_3_0_mm_3D_glrlm_LongRunEmphasis
10
5
0
-5
-10
cancer
1
0
nodule
1
0
incident_lung
0
1
family_history_LC_firstdegree
1
0
diabetes
1
0
hyperlipemia
1
0
hypertension
1
0
chronic_respiratory_disease
1
0
cancer_history
1
0
cluster
1
2

(d) LL

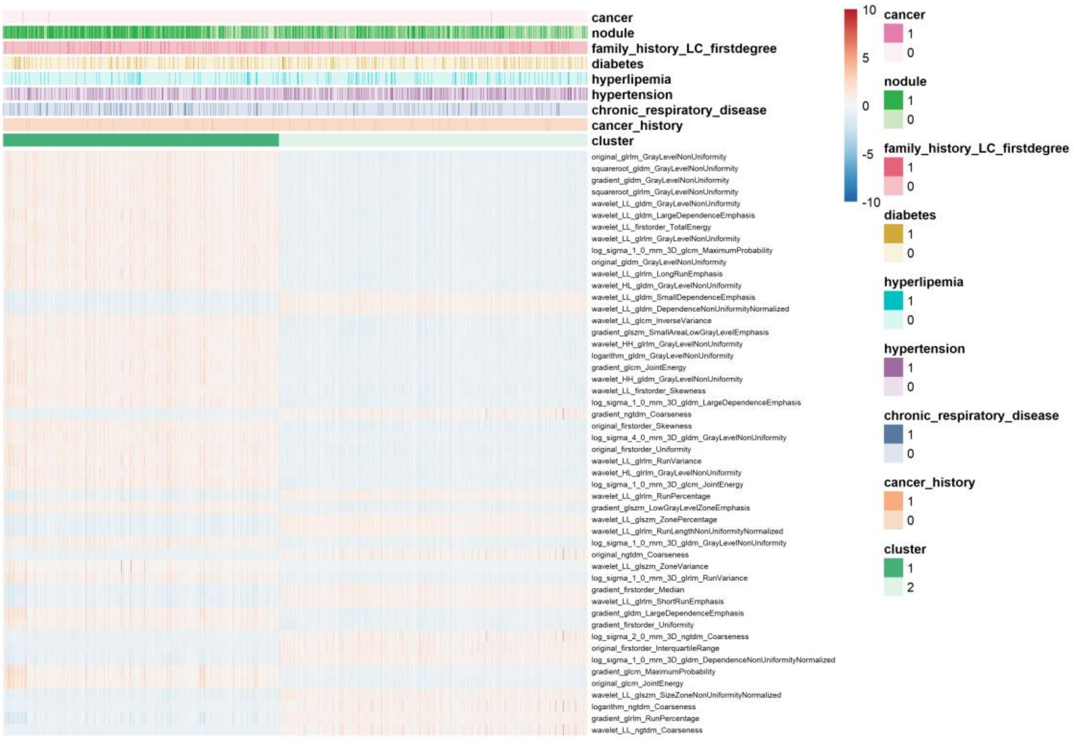
cancer
nodule
family_history_LC_firstdegree
diabetes
hyperlipemia
hypertension
chronic_respiratory_disease
cancer_history
cluster
original_glrlm_GrayLevelNonUniformity
squareroot_gldm_GrayLevelNonUniformity
gradient_gldm_GrayLevelNonUniformity
squareroot_glrlm_GrayLevelNonUniformity
wavelet_LL_gldm_GrayLevelNonUniformity
wavelet_LL_gldm_LargeDependenceEmphasis
wavelet_LL_firstorder_TotalEnergy
wavelet_LL_glrlm_GrayLevelNonUniformity
log_sigma_1_0_mm_3D_glcm_MaximumProbability
original_gldm_GrayLevelNonUniformity
wavelet_LL_glrlm_LongRunEmphasis
wavelet_HL_gldm_GrayLevelNonUniformity
wavelet_LL_gldm_SmallDependenceEmphasis
wavelet_LL_gldm_DependenceNonUniformityNormalized
wavelet_LL_glcm_InverseVariance
gradient_glszm_SmallAreaLowGrayLevelEmphasis
wavelet_HH_glrlm_GrayLevelNonUniformity
logarithm_gldm_GrayLevelNonUniformity
gradient_glcm_JointEnergy
wavelet_HH_gldm_GrayLevelNonUniformity
wavelet_LL_firstorder_Skewness
log_sigma_1_0_mm_3D_gldm_LargeDependenceEmphasis
gradient_ngtdm_Coarseness
original_firstorder_Skewness
log_sigma_4_0_mm_3D_gldm_GrayLevelNonUniformity
original_firstorder_Uniformity
wavelet_LL_glrlm_RunVariance
wavelet_HL_glrlm_GrayLevelNonUniformity
log_sigma_1_0_mm_3D_glcm_JointEnergy
wavelet_LL_glrlm_RunPercentage
gradient_glszm_LowGrayLevelZoneEmphasis
wavelet_LL_glszm_ZonePercentage
wavelet_LL_glrlm_RunLengthNonUniformityNormalized
log_sigma_1_0_mm_3D_gldm_GrayLevelNonUniformity
original_ngtdm_Coarseness
wavelet_LL_glszm_ZoneVariance
log_sigma_1_0_mm_3D_glrlm_RunVariance
gradient_firstorder_Median
wavelet_LL_glrlm_ShortRunEmphasis
gradient_gldm_LargeDependenceEmphasis
gradient_firstorder_Uniformity
log_sigma_2_0_mm_3D_ngtdm_Coarseness
original_firstorder_InterquartileRange
log_sigma_1_0_mm_3D_gldm_DependenceNonUniformityNormalized
gradient_glcm_MaximumProbability
original_glcm_JointEnergy
wavelet_LL_glszm_SizeZoneNonUniformityNormalized
logarithm_ngtdm_Coarseness
gradient_glrlm_RunPercentage
wavelet_LL_ngtdm_Coarseness
10
5
0
-5
-10
cancer
1
0
nodule
1
0
family_history_LC_firstdegree
1
0
diabetes
1
0
hyperlipemia
1
0
hypertension
1
0
chronic_respiratory_disease
1
0
cancer_history
1
0
cluster
1
2
